# Sustainable development and women’s empowerment in vulnerable communities: an experience report

**DOI:** 10.1590/0034-7167-2024-0335

**Published:** 2025-09-01

**Authors:** Ana Paula Rodrigues Arciprete, Rosa Vanessa Alves Lima, Tatiane Marçal Silva Cardoso, Flávia Gomes-Sponholz, Juliana Cristina dos Santos Monteiro

**Affiliations:** I Universidade de São Paulo. Ribeirão Preto, São Paulo, Brazil

**Keywords:** Empowerment, Vulnerable Population, Women’s Health, Sustainable Development, Community Health Nursing., Empoderamiento, Comunidades Vulnerables, Salud de la Mujer, Desarrollo Sostenible, Enfermería en Salud Comunitaria.

## Abstract

**Objectives:**

to describe the development and implementation of a project aimed at empowering entrepreneurial women in vulnerable communities.

**Methods:**

this experience report details an extension project conducted in Ribeirão Preto, São Paulo, Brazil, in 2021. The project was a collaboration among nursing faculty and students, community leaders, and public and private institutions.

**Results:**

a mapping process gathered information to produce a situational diagnosis of the women entrepreneurs, who participated in workshops on economics, business management, and health. The collaborative network facilitated the legal establishment of a cooperative of seamstresses. The project demonstrated transformative potential by fostering engagement in vulnerable communities and exposing students to issues that significantly affect well-being and quality of life, particularly for women.

**Conclusions:**

the project’s implementation provides a foundation for future health professionals to act critically and proactively, focusing on human and social dimensions and contributing to the effective achievement of the Sustainable Development Goals.

## RESUMEN

**Objetivos:**

describir la elaboración y ejecución de un proyecto de empoderamiento de mujeres emprendedoras en comunidades vulnerables.

**Métodos:**

relato de experiencia de un proyecto de extensión desarrollado en Ribeirão Preto/SP, en 2021, mediante la colaboración entre docentes y estudiantes de Enfermería, líderes comunitarios e instituciones públicas y privadas.

**Resultados:**

el mapeo inicial proporcionó información para el diagnóstico situacional de mujeres emprendedoras, quienes participaron en talleres sobre economía, gestión de negocios y salud. La red colaborativa facilitó la formalización legal de una cooperativa de costureras. El proyecto demostró un potencial transformador tanto en la intervención en comunidades vulnerables como en la aproximación de estudiantes a temas que impactan el bienestar y la calidad de vida, especialmente de mujeres.

**Conclusiones:**

el proyecto provee herramientas para que futuros profesionales de salud actúen de forma crítica y proactiva, promoviendo la dimensión humana y social y contribuyendo al cumplimiento de los Objetivos de Desarrollo Sostenible.

## INTRODUCTION

The integration of women into the labor market, often in full-time positions, combined with their responsibilities in managing household and childcare activities, frequently results in a double or even triple workload, leading to overburdening and exhaustion. However, formal employment opportunities for women have contributed to increase household income and, in many cases, have served as a tool for women’s empowerment^(1)^.

In this context, fostering both individual and collective capacities is crucial to enabling women to actively and effectively participate in the productive chain, equipped with the necessary skills and leadership qualities to perform various activities. Developing internal attributes such as self-esteem and self-confidence is fundamental, empowering women to take an active role in shaping this process^(2)^.

Building robust economies that support women’s full participation across economic sectors is essential. This objective is achieved within a framework of greater justice and social equity by promoting women’s empowerment and implementing policies and action programs that uphold sustainability and human rights, thereby improving the quality of life for all^(3)^.

In Ribeirão Preto, São Paulo state, women who manage small businesses within their communities drive numerous initiatives. Due to the country’s current health and political climate, women seeking to support their families through entrepreneurial activities started many ventures. In the northern region of Ribeirão Preto, one of the neighborhoods characterized by social vulnerability, women collaborate on collective ventures such as community kitchens, sewing cooperatives, clothing sales, and beauty services, among other entrepreneurial initiatives. Despite the broader availability of courses in entrepreneurship and greater access to financial credit being recognized as beneficial for small business owners^(4)^, these women’s initiatives often fail to establish themselves as sustainable enterprises or primary income sources. Many face limited access to investment opportunities, such as financing for their establishments, or lack the administrative knowledge required for effective business management.

In 2015, the United Nations (UN) launched the Sustainable Development Goals (SDGs) to be achieved by 2030, aiming to promote equitable, inclusive, and sustainable social development while addressing economic and environmental considerations on a global scale^(5)^.

Aligned with these goals, we focused on “Goal 11 - Sustainable Cities and Communities: Make cities and human settlements inclusive, safe, resilient, and sustainable”^(5)^ to develop the outreach project “Female entrepreneurship in vulnerable communities of Ribeirão Preto/SP: actions for empowerment and sustainability”. This project aimed to equip women from participating communities with skills to manage their entrepreneurial initiatives, thereby increasing the sustainability of their economic activities while empowering women and strengthening their businesses within their communities.

The project was part of the “USP Municípios Program”, which encourages the development of projects to address municipal challenges in São Paulo state, focusing on achieving Goal 11 of the SDGs^(6)^. The “USP Challenge: Sustainable Cities” call for proposals engaged undergraduate and graduate students in developing projects featuring actionable strategies for municipalities to implement. These initiatives contributed to achieving the targets under Goal 11 in areas such as safe, accessible, and sustainable transportation, energy efficiency, smart cities, waste management, access to safe public spaces, housing, sustainable urban agriculture, and policies for empowering women and girls, as well as other groups facing heightened vulnerability in the labor market^(6)^. In addition to Goal 11, the project indirectly addressed and supported the achievement of Goal 1 - End poverty in all its forms everywhere, Goal 3 - Ensure healthy lives and promote well-being for all at all ages, and Goal 5 - Achieve gender equality and empower all women and girls.

Thus, the aforementioned outreach project aimed to train women in the involved communities to manage their entrepreneurial initiatives, enhancing their ability to sustain these economic activities. It also sought to empower them and strengthen their ventures within their communities. Considering these points, this article aims to highlight this topic and explore new opportunities for nursing practice to contribute to achieving the SDGs.

## OBJECTIVES

To report the experience of designing and implementing an extension project developed by faculty members and students from a public nursing school in the interior of São Paulo state. The project was conducted with women entrepreneurs from socially vulnerable communities in the municipality, highlighting the facilitators and challenges encountered during its implementation.

## METHODS

This study reports on the experience of designing and implementing the extension project titled *“*Female entrepreneurship in vulnerable communities of Ribeirão Preto, São Paulo state: actions for empowerment and sustainability”. The Santander Public Policy Program - USP Sustainable Cities Challenge supported the project, which community leaders and public and private institutions implemented through partnerships.

The project adhered to national and international ethical guidelines. As an experience report, it did not require approval from an ethics committee or the inclusion of an Informed Consent Form (ICF).

The implementation team consisted of seven scholarship recipients, including two postgraduate nurses and five undergraduate nursing students, under the coordination and mentorship of two faculty members. As outlined below, we developed the project in stages to achieve its objectives.

Stage 1 - Planning (14 days):

1. Weekly Meetings: Conducted among mentors, scholarship recipients, community leaders, partners, and the participating women;
2. Mapping: Documented women entrepreneurs and their businesses in participating communities using data from community leaders, family registries, and local business activity reports. To ensure accurate territorial representation, we conducted four visits to the communities, which comprise approximately 150 vulnerable families;
3. Needs Assessment: Identified the primary demands of the women entrepreneurs regarding their businesses;
4. Vulnerability Identification: Mapped women entrepreneurs experiencing heightened vulnerability;
5. Result Sharing: Presented the mapping results during a meeting with community leaders and local women.

To transition between “Stage 1 - Planning” and “Stage 2 - Execution”, the team organized meetings to present the proposal to three potential partners: a non-governmental organization supporting women in one of the communities, a collective active in two other communities, and selected community leaders. While one community leader opted out due to similar ongoing initiatives, the leaders from the other two communities readily agreed to participate. Notably, these communities are geographically close, and their leaders and collective members often collaborate on various activities.

Stage 2 - Execution:

1. Partnership Development: Collaborated with the Brazilian Support Service for Micro and Small Enterprises (SEBRAE) and two university professors specializing in solidarity economy to develop training activities on entrepreneurship and business management;
2. Workshops: Conducted workshops on business management, health, and well-being led by partners and scholarship recipients, tailored to the needs identified by the women entrepreneurs;
3. Accounting Support: Established a partnership with an accountant who voluntarily provided group and individual consultations to the women entrepreneurs;
4. Network Building: Facilitated partnerships with two clothing companies for the cooperative of seamstresses to produce garments.

Throughout the project implementation, from August to December 2021, weekly meetings were held between project representatives (scholarship recipients and mentors), community leaders, partners, and the participating women to organize activities.

During these activities, we assessed the community’s geographic space, infrastructure, and accessibility while cataloging local businesses based on family registries. Using this data, the scholarship recipients created maps and reports detailing the dynamics of the women entrepreneurs’ lives and their businesses within the community.

To plan, organize, and conduct the training sessions, we identified partners to provide content aligned with the needs identified during the mapping process. We scheduled training dates based on the availability shared by the women during weekly meetings. These activities were promoted via WhatsApp^®^ using messages and digital flyers created by the scholarship recipients.

## THE EXPERIENCE, ITS RESULTS, AND COMMUNITY IMPACT

We conducted a community mapping to gather information for a situational diagnosis, which we shared during a weekly meeting. From this, we proposed selecting ten entrepreneurs to participate in training sessions based on their level of social vulnerability and identified priorities. However, the women collectively decided that it was important for all interested individuals to join the training sessions. This decision stemmed from the community’s plan to establish a formal seamstress cooperative, recognizing that these training workshops could enhance and strengthen entrepreneurial efforts for all participants. We promptly honored their request.

Between October and December 2021, six workshops were held biweekly, involving 29 women and covering the following topics: “Simplify: Business Formalization” (offered in partnership with SEBRAE); and “Solidarity Economy”, “Women’s Health and Entrepreneurship”, “Addressing Domestic Violence”, “Family Planning”, “Business Models”, and “E-commerce” (provided by the project team and partnering faculty).

In addition to the workshops, which were the project’s primary focus for empowering the women, collaborative efforts between community leaders and project fellows created a robust network of contacts that amplified the project’s visibility and impact within the municipality. This outreach sparked interest from two clothing companies in Ribeirão Preto, which sought to contract the seamstress cooperative-then in the process of creation and formalization-for garment production services. In October 2021, representatives from both companies visited the future cooperative headquarters to discuss partnership details, a development celebrated by all involved women.

Another significant project outcome was a partnership with an accountant who volunteered to provide group and individual advisory services to the women entrepreneurs and the seamstress cooperative.

The project concluded with a group evaluation session involving the tutor, project fellows, community leaders, and participants. During this session, we learned that the cooperative’s formalization process was underway, with documents being gathered to secure a corporate taxpayer registration number (CNPJ). Participants shared that the mapping and workshops positively influenced both individual and group entrepreneurial attitudes, as they boosted self-esteem, encouraged self-care, and inspired further efforts to promote entrepreneurship within their communities.

Currently, university extension activities are undergoing a process of integration into curricula to align with the 2014-2024 National Education Plan. This plan mandates that at least 10% of the undergraduate curriculum should include extension activities, fostering an effective relationship between the university and the community^(7)^. These activities are expected to address emerging social demands and seek innovative, impactful solutions, enabling students to broaden their experiences for improved professional development and a deeper integration of theory and practice^(7)^.

This project presented both challenges and transformative potential, particularly in its work with socially vulnerable communities and its ability to expose health students to issues that directly affect well-being and quality of life, especially for women. The undergraduate fellows and postgraduate volunteers also worked diligently to disseminate this work within academic environments by presenting preliminary results at scientific events.

In this context, healthcare professionals must be prepared for innovative and creative social interventions. They should embrace their roles as social agents with personal responsibility, adopting a perspective that fosters citizenship and transcends disciplinary knowledge and institutional roles^(8)^. The project team took this approach, engaging nursing students in extramural activities that positively impacted the women participating in these initiatives.

Based on the needs identified during the initial mapping, the training sessions on business management and health education proved effective in illuminating the general living conditions of the entrepreneurs in the participating communities. Overall, this project met its objectives and contributed to achieving Goal 11 of the SDGs.

Additionally, this project has the potential to positively influence environmental impact reduction and strengthen economic, social, and environmental connections between urban and peri urban areas. The workshops and activities raised awareness and provided training for women in sustainable business practices, emphasizing solidarity economy and cooperation.

Moreover, this project encouraged reflection on the role of nursing in fostering social emancipation through the promotion of holistic health, which supports well-being and is directly tied to an improved quality of life. In this regard, a study conducted during the COVID-19 pandemic involving women who worked as recyclable material collectors highlighted the significance of community extension activities^(9)^. In that study, health education actions in partnership with community leaders resulted in recognizing and appreciating women often marginalized and rendered invisible in their work. Furthermore, these actions demonstrated that nursing offers numerous opportunities for students in training, paving the way for discussions on nursing entrepreneurship, social entrepreneurship, and innovation^(9)^.

### Study limitations

We encountered several challenges during the project’s development, including difficulty accessing project locations, establishing and strengthening bonds with local leaders and the community due to the short execution period, and navigating barriers in coordination with leaders and partner institutions. These obstacles were tied to the political and infrastructural dynamics of the communities, which involve ongoing territorial disputes. However, these limitations were successfully addressed, as evidenced by the promising results and the positive feedback from participants.

### Contributions to the Field

This project stands out for its pioneering approach to engaging nursing faculty, undergraduate, and graduate students in fieldwork to understand and contribute to women entrepreneurs’ life, health, and well-being dynamics in socially vulnerable communities. The work also highlights the critical role and representation of nursing in achieving the SDGs. Nurses, who form the largest contingent of the healthcare workforce, play a fundamental role in advancing science, technology, and innovations in public health policies^(10)^.

## CONCLUSIONS

Based on the findings, we conclude that the project’s objectives were fully achieved, with results surpassing expectations. The workshops significantly contributed to establishing the seamstresses’ cooperative, while the collaborative network facilitated the implementation of income generating proposals for women in the communities.

Recognizing women holistically and viewing health care as a means of empowerment can impact their quality of life and contribute to sustaining and advancing their businesses.

At the municipal public policy level, this proposal can be maintained, refined, and adapted for other extension projects, with women themselves serving as multipliers to train new groups. Additionally, it is replicable for other communities and could be integrated into the municipality’s Departments of Innovation and Development and Social Assistance.

The challenges and opportunities highlighted during the project’s development underscore the importance of nurses and other healthcare professionals in promoting comprehensive health, fostering well-being, and supporting social emancipation, all directly linked to improving quality of life. This project provides a foundation for future healthcare professionals to act critically and proactively as social entrepreneurs, engaging systematically with a focus on human, social, and sustainable dimensions, thereby contributing to the effective realization of the SDGs.
