# Supplementary material for: Sustainable development and women’s empowerment in vulnerable communities: an experience report
Source: Rev Bras Enferm. 2025 Sep 1;78(Suppl 1):e20240335. doi: 10.1590/0034-7167-2024-0335 (PMC12407881; doi:10.1590/0034-7167-2024-0335)
Supplement: Translation

# Translation: Desenvolvimento sustentável e empoderamento feminino em comunidades vulnerabilizadas: um relato de experiência

**DOI:** 10.1590/0034-7167-2024-0335pt

**Authors:** Ana Paula Rodrigues Arciprete, Rosa Vanessa Alves Lima, Tatiane Marçal Silva Cardoso, Flávia Gomes-Sponholz, Juliana Cristina dos Santos Monteiro

**Affiliations:** I Universidade de São Paulo. Ribeirão Preto, São Paulo, Brasil

**Keywords:** Empoderamento, Comunidades Vulneráveis, Saúde da Mulher, Desenvolvimento Sustentável, Enfermagem em Saúde Comunitária.

## Abstract

**Objetivos:**

relatar a elaboração e execução de projeto para empoderamento de mulheres empreendedoras de comunidades vulnerabilizadas.

**Métodos:**

relato de experiência do projeto de extensão desenvolvido em Ribeirão Preto/SP, em 2021, mediante parceria entre docentes e discentes de Enfermagem, lideranças comunitárias, instituições públicas e privadas.

**Resultados:**

um mapeamento levantou informações para o diagnóstico situacional das mulheres empreendedoras, que participaram de oficinas sobre economia, gestão de negócios e saúde. A rede de colaboração possibilitou a tramitação legal de uma cooperativa de costureiras. O projeto mostrou potencial transformador tanto na atuação em comunidades vulnerabilizadas quanto na aproximação de estudantes a questões que geram impacto no bem-estar e qualidade de vida, principalmente de mulheres.

**Conclusões:**

a execução do projeto fornece subsídios para que futuros profissionais de saúde atuem de forma crítica e proativa com foco na dimensão humana e social, contribuindo para o alcance efetivo dos Objetivos de Desenvolvimento Sustentável.

## INTRODUÇÃO

A inserção das mulheres no mercado de trabalho, muitas vezes em ocupações com jornada integral, juntamente com o exercício das atividades de cuidado do lar e dos filhos acarretam dupla ou tripla jornada de trabalho, gerando a elas sobrecarga e exaustão. Contudo, o mercado de trabalho formal para mulheres possibilitou o aumento na renda familiar e, em muitos casos, tornou-se uma ferramenta de empoderamento feminino^(1)^.

Nesse sentido, a atuação nas capacidades individuais e coletivas se faz necessária, a fim de que elas se insiram efetivamente na cadeia produtiva com capacitação e liderança e executem diversas atividades. Para isso, é fundamental desenvolver habilidades internas como autoestima e autoconfiança, de modo que possam ser ativas e construtoras desse processo^(2)^.

É necessário construir economias fortes para favorecer a participação integral de mulheres nos setores econômicos. Essa meta é alcançada dentro de um contexto de maior justiça e igualdade social por meio do empoderamento feminino e desenvolvimento de políticas e programas de ação que respeitem a sustentabilidade e os direitos humanos. Dessa forma, contribui-se para a melhoria da qualidade de vida a todos^(3)^.

No município de Ribeirão Preto, estado de São Paulo, observam-se diversas frentes de trabalho de mulheres com pequenos empreendimentos organizados dentro de comunidades. Por causa da atual conjuntura sanitária e política do país, muitos desses empreendimentos foram criados por mulheres que tentam sustentar suas famílias por meio de seus negócios. Na zona norte de Ribeirão Preto, em um dos bairros que abrange comunidades em situação de vulnerabilidade social, as mulheres se unem para movimentar empreendimentos coletivos, como cozinha para alimentação comunitária, cooperativa de costura, venda de roupas e serviços de beleza, entre outras iniciativas empreendedoras. Embora a difusão mais ampla de cursos na área de empreendedorismo e a maior disponibilidade de crédito financeiro sejam vistos como benéficos para alavancar pequenos empreendedores^(4)^, nem sempre as iniciativas dessas mulheres conseguem firmar-se de forma duradoura ou como única fonte de renda. Isso porque muitas delas não têm acesso a possibilidades de investimento, como financiamento para seus estabelecimentos ou mesmo saberes administrativos para gestão de negócios.

Em 2015, a Organização das Nações Unidas (ONU) lançou os Objetivos de Desenvolvimento Sustentável (ODS), para serem alcançados até 2030. Eles visam à promoção do desenvolvimento social equitativo, inclusivo e sustentável, considerando os aspectos econômicos e ambientais em escala global^(5)^.

Nesse sentido, tomamos como de interesse o “Objetivo 11 - Cidades e comunidades sustentáveis: tornar cidades e assentamentos humanos mais inclusivos, seguros, resilientes e sustentáveis”^(5)^ ao desenvolvermos o projeto de extensão “Empreendedorismo feminino em comunidades vulneráveis de Ribeirão Preto/SP: ações para empoderamento e sustentabilidade”. Esse projeto objetivou capacitar as mulheres das comunidades envolvidas para a gestão de suas iniciativas empreendedoras, favorecendo maiores possibilidades de manter suas atividades econômicas e contribuindo para empoderar as mulheres e fortalecer seus empreendimentos dentro das comunidades.

O projeto fez parte do “Programa USP Municípios”, que visa estimular o desenvolvimento de projetos para solucionar problemas dos municípios do estado de São Paulo mediante o foco no Objetivo 11 dos ODS^(6)^. O edital “Desafio USP: Cidades Sustentáveis” envolveu estudantes de graduação e pós-graduandos no desenvolvimento de projetos com ações e estratégias possíveis de implementação pelas prefeituras. Dessa maneira, contribuiu para atingir as metas estabelecidas nesse Objetivo em áreas como transporte seguro, acessível e sustentável, eficiência energética, cidades inteligentes, gestão de resíduos, acesso a espaços públicos seguros, moradia, agricultura urbana sustentável e políticas de empoderamento de meninas e mulheres bem como de grupos com maior vulnerabilidade no mercado de trabalho^(6)^. Somado ao Objetivo 11, indiretamente o projeto também contemplou e colaborou para o alcance dos Objetivos 1 - Erradicação da pobreza, 3 - Saúde e bem estar e 5 - Igualdade de gênero.

Dessa maneira, o projeto de extensão citado visou capacitar as mulheres das comunidades envolvidas para a gestão de suas iniciativas empreendedoras. Ele favorece maiores possibilidades de manter tais atividades econômicas e contribui para empoderá-las e fortalecer seus empreendimentos dentro das comunidades. Considerando o exposto, a elaboração deste artigo tem o objetivo de dar visibilidade ao tema e apontar novas possibilidades para a atuação da enfermagem no alcance dos ODS.

## OBJETIVOS

Relatar a experiência da elaboração e execução de um projeto de extensão desenvolvido por docentes e discentes de uma faculdade pública de Enfermagem do interior do estado de São Paulo com mulheres empreendedoras de comunidades vulnerabilizadas desse município, apresentando as facilidades e os desafios na sua implementação.

## MÉTODOS

Trata-se de um relato de experiência sobre a elaboração e execução do projeto de extensão intitulado “Empreendedorismo feminino em comunidades vulneráveis de Ribeirão Preto/SP: ações para empoderamento e sustentabilidade”. Foi fomentado pelo Programa Santander de Políticas Públicas - Desafio USP Cidades Sustentáveis e implementado por intermédio de parcerias com lideranças comunitárias e instituições públicas e privadas locais.

O projeto foi conduzido de acordo com as diretrizes de ética nacionais e internacionais. Por ser um relato de experiência, ele dispensa a aprovação por comitê de ética e a inclusão de Termo de Consentimento Livre e Esclarecido.

A equipe de execução foi composta por sete bolsistas, sendo duas enfermeiras pós-graduandas e cinco graduandas em Enfermagem, sob a coordenação e tutoria de duas docentes. Para alcançar os objetivos propostos, o projeto foi desenvolvido em etapas, descritas a seguir.

Etapa 1 - Planejamento (duração 14 dias):

1. Realização de reuniões semanais entre tutores, bolsistas, lideranças comunitárias, parceiros e mulheres envolvidas;
2. Mapeamento das mulheres empreendedoras e de seus empreendimentos, nas comunidades participantes, tendo como base o cadastro das famílias moradoras e as informações sobre as atividades de negócios locais, fornecidos pelas lideranças das comunidades. Para que o mapeamento fosse fidedigno ao território, foram realizadas quatro visitas às comunidades, onde vivem cerca de 150 famílias vulnerabilizadas;
3. Identificação das principais demandas das mulheres das comunidades sobre seus empreendimentos;
4. Identificação das mulheres empreendedoras em situação de vulnerabilidade;
5. Divulgação dos resultados do mapeamento em reunião com as lideranças comunitárias e mulheres das comunidades.

Como transição entre a “Etapa 1 - Planejamento” e a “Etapa 2 - Execução”, foram realizadas reuniões para apresentar a proposta a três possíveis parceiros: uma organização não governamental que assiste mulheres em uma das comunidades; um coletivo que atua em outras duas comunidades; e as lideranças comunitárias elencadas para receberem as ações do projeto. Uma das lideranças da comunidade optou por não participar do projeto por já estarem realizando trabalho semelhante com outros parceiros. Os líderes das outras duas comunidades prontamente concordaram em participar. Vale destacar que essas comunidades são muito próximas, e as lideranças e membros do coletivo realizam várias atividades conjuntamente.

Etapa 2 - Execução:

1. Estabelecimento de parcerias com o Serviço Brasileiro de Apoio às Micro e Pequenas Empresas (SEBRAE) e com duas docentes universitárias atuantes na área de economia solidária, visando desenvolver as atividades de capacitação sobre empreendedorismo e gestão de negócios;
2. Realização de oficinas sobre gestão de negócios e saúde/bem-estar oferecidas pelos parceiros e bolsistas às mulheres empreendedoras com base nas demandas apontadas pelas próprias mulheres;
3. Estabelecimento de parceria com uma contadora que realizou assessoria em grupo e individual de forma voluntária às mulheres empreendedoras;
4. Articulação da rede local por meio de parceria com duas empresas de vestuário para confecção de roupas pela cooperativa de costureiras da comunidade.

Ao longo dos meses de execução do projeto (de agosto a dezembro de 2021) foram realizadas reuniões semanais entre representantes do projeto (bolsistas e tutoras) e das comunidades (líderes e parceiros) e com as próprias mulheres para organização das atividades.

Assim, foram realizados o reconhecimento do espaço geográfico da comunidade, sua infraestrutura e formas de acesso e o levantamento dos empreendimentos locais com base no cadastro das famílias moradoras. Com essas informações, as bolsistas do projeto elaboraram mapas e relatórios das visitas, identificando como se dá a dinâmica de vida tanto das mulheres empreendedoras na comunidade quanto de seus empreendimentos.

Para o planejamento, organização e realização das capacitações, prospectamos parceiros que oferecessem conteúdos de acordo com as demandas identificadas no mapeamento. O agendamento das datas para as capacitações foi realizado conforme disponibilidade informada pelas mulheres nas reuniões semanais. A divulgação dessas atividades foi feita por meio do *WhatsApp* ^®^ com o uso de mensagens e folders digitais criados pelas bolsistas.

## A EXPERIÊNCIA, SEUS RESULTADOS E O IMPACTO NA COMUNIDADE

O mapeamento das comunidades levantou informações que permitiram o diagnóstico situacional, sendo divulgadas em reunião semanal. A partir daí, propusemos a atividade seguinte, que seria a seleção de dez empreendedoras para receberem capacitações de acordo com a vulnerabilidade social e as prioridades das mulheres envolvidas. No entanto, elas próprias decidiram que seria importante todas as interessadas participarem das capacitações. Isso porque a comunidade estava, naquele momento, planejando criar uma cooperativa formal de costureiras, de modo que as oficinas de capacitação poderiam contribuir para o aprimoramento e fortalecimento de todas para ações empreendedoras. Essa solicitação das mulheres foi prontamente atendida.

De outubro a dezembro de 2021, foram realizadas seis oficinas a cada 15 dias, as quais atingiram 29 mulheres e apresentaram as seguintes temáticas: Descomplique: Formalização de Empresas (oferecida em parceria com o SEBRAE); e Economia Solidária, Saúde das Mulheres e empreendedorismo, Enfrentamento da Violência Doméstica, Planejamento Familiar, Modelo de Negócios e E-commerce (oferecidas pela equipe do projeto e docentes parceiros).

Além das oficinas realizadas, foco principal deste projeto para fortalecimento das mulheres, a articulação das líderes comunitárias e bolsistas em trabalhos colaborativos e formação de rede de contatos possibilitou maior visibilidade e repercussão desse projeto no município. Isso culminou com o interesse de duas empresas de vestuário de Ribeirão Preto em contratar o serviço de confecção de roupas da cooperativa de costureiras que estava sendo criada e formalizada por mulheres das comunidades. Em outubro de 2021, representantes das duas empresas visitaram o espaço da futura sede da cooperativa para as negociações relativas a essa parceria, o que foi um resultado bastante comemorado pelas mulheres envolvidas.

Outro resultado alcançado por esse projeto, graças à projeção das ações no município, foi o estabelecimento de parceria com uma contadora que se ofereceu voluntariamente para prestar a assessoria em grupo e individual às mulheres empreendedoras e à cooperativa de costureiras.

As atividades do projeto foram finalizadas com uma avaliação em grupo feita pela tutora, bolsistas, líderes comunitárias e mulheres. Nesse dia, fomos informadas de que o estabelecimento da cooperativa de costureiras estava em processo de tramitação, com o levantamento de documentos para aquisição de CNPJ. As participantes manifestaram que o mapeamento e oficinas realizados pelo projeto influenciaram positivamente a atitude empreendedora individual e grupal, pois proporcionaram aumento da autoestima e autocuidado bem como estimularam e impulsionaram os esforços para o empreendedorismo nas comunidades.

Na atualidade, as atividades de extensão universitária passam por um processo de curricularização visando atender ao Plano Nacional de Educação 2014-2024, o qual preconiza que ao menos 10% da carga horária curricular da graduação deve ser realizada com ações extensionistas, para possibilitar a relação efetiva entre a universidade e a comunidade^(7)^. Essas ações devem suprir as demandas sociais emergentes assim como compreender e buscar soluções inovadoras e de impacto, possibilitando aos graduandos a ampliação de suas vivências para um melhor desenvolvimento profissional e integração entre teoria e prática^(7)^.

Dessa forma, o projeto apresentou um desafio e um potencial transformador tanto na atuação em comunidades em situação de vulnerabilidade quanto na aproximação de estudantes da área da saúde a questões que geram impacto direto no bem-estar e qualidade de vida, principalmente de mulheres. Vale destacar que as bolsistas graduandas e voluntárias pós-graduandas também envidaram esforços para a divulgação desse trabalho em ambiente acadêmico mediante a apresentação de resultados preliminares em eventos científicos.

Nesse sentido, considerando a prática dos profissionais de saúde, estes devem estar preparados para intervenções sociais inovadoras e criativas. Devem assumir seu papel como atores sociais com responsabilidades pessoais, numa atitude voltada para o desenvolvimento da cidadania e extrapolação dos saberes disciplinares e papéis institucionais^(8)^. Esta foi a postura da equipe participante do projeto, que se dispôs a levar as estudantes de Enfermagem para a realização de atividades extramuros da universidade, causando impactos positivos nas mulheres atendidas pelas ações.

De acordo com as necessidades identificadas pelo mapeamento inicial, as atividades de capacitação sobre gestão de negócios e educação em saúde para as mulheres se mostraram efetivas para expor o quadro geral de vida das empreendedoras das comunidades participantes. De forma geral, o presente trabalho atingiu seus objetivos e contribuiu para o alcance do Objetivo 11 dos ODS.

Adicionalmente, este projeto tem o potencial de auxiliar positivamente na redução do impacto ambiental e no fortalecimento das relações econômicas, sociais e ambientais entre áreas urbanas e periurbanas, visto que as oficinas e atividades envolveram a sensibilização e capacitação de mulheres em práticas sustentáveis para seus negócios, com destaque para a economia solidária e cooperação.

Para além, este projeto possibilitou a reflexão sobre o papel da enfermagem na busca pela emancipação social por meio da promoção da saúde integral, que favorece o bem-estar e está diretamente ligada à melhor qualidade de vida. Nesse sentido, um estudo desenvolvido durante a pandemia da covid 19 com mulheres que atuavam recolhendo materiais recicláveis destacou a importância das atividades de extensão à comunidade^(9)^. Isso porque, nesse estudo mencionado, as ações educativas em saúde realizadas em parceria com líderes comunitárias geraram reconhecimento e valorização das mulheres que, frequentemente, são marginalizadas e invisibilizadas em suas atividades laborais. Também, tais ações mostraram que a enfermagem pode oferecer inúmeras possibilidades aos alunos em formação, abrindo espaço para a discussão sobre empreendedorismo em enfermagem, empreendedorismo social e inovação^(9)^.

### Limitações do estudo

Algumas barreiras foram encontradas para o desenvolvimento do projeto, tais como: dificuldade de acesso aos locais; dificuldade em estruturar e fortalecer o vínculo com as lideranças locais e com a própria comunidade em razão do curto período de execução do projeto; e entraves na articulação com as lideranças e instituições parceiras devido à dinâmica de estabelecimento dessas comunidades, que envolve questões políticas e infraestruturais bem como permanente disputa territorial. No entanto, essas limitações foram superadas haja vista os resultados promissores e a avaliação positiva das envolvidas.

### Contribuições para a Área

É importante destacar o pioneirismo do projeto, ao buscar realizar uma atividade de extensão que leve docentes junto com alunas de graduação e pós-graduação em Enfermagem a campo para compreender e contribuir com a dinâmica de vida, saúde e bem-estar das mulheres empreendedoras de comunidades vulnerabilizadas. Além disso, o trabalho realça a relevância e representatividade da enfermagem para o alcance dos ODS, já que essa categoria representa o maior contingente da força de trabalho da saúde, tendo um papel fundamental em fomentar ciência, tecnologias e inovações para o campo das políticas públicas de saúde^(10)^.

## CONCLUSÕES

Com base no exposto, considera-se que todos os objetivos do projeto foram atingidos e os resultados alcançados foram além das expectativas, visto que as oficinas contribuíram fortemente para o estabelecimento da cooperativa de costureiras e a articulação em rede permitiu a efetivação de propostas para gerar renda às mulheres das comunidades.

A compreensão dessas mulheres em sua integralidade e da própria atenção à saúde como fatores capazes de favorecer o empoderamento pode gerar impacto real na qualidade de vida dessas pessoas e contribuir para a manutenção e impulsionamento de seus negócios.

Considerando a implementação de políticas públicas municipais, a proposta pode ser mantida, aperfeiçoada e utilizada por outros projetos de extensão mediante a organização das próprias mulheres como multiplicadoras para a capacitação de novos grupos. Além disso, é replicável para outras comunidades e pode ser absorvida pelas Secretarias de Inovação e Desenvolvimento e de Assistência Social do município.

Dessa forma, os desafios e potencialidades expostos no desenvolvimento do projeto evidenciam a importância de enfermeiros e de outros profissionais de saúde para a promoção da saúde integral na busca pelo bem-estar e emancipação social, o que está diretamente relacionado à melhoria da qualidade de vida das pessoas. O relato deste projeto fornece subsídios para que os futuros profissionais da saúde sejam capazes de agir, crítica e proativamente, como empreendedores sociais em atuação sistêmica com foco na dimensão humana, social e sustentável, contribuindo, assim, para o alcance efetivo dos ODS.
